# Chitosan-Based Gastric Dressing Materials Loaded with Pomegranate Peel as Bioactive Agents: Pharmacokinetics and Effects on Experimentally Induced Gastric Ulcers in Rabbits

**DOI:** 10.3390/metabo12121158

**Published:** 2022-11-22

**Authors:** Samira Jebahi, Ghada Ben Salah, Soufien Jarray, Mounir Naffati, Mohammad Ayaz Ahmad, Faten Brahmi, Mohd Saeed, Arif J. Siddiqui, Khabir Abdelmajid, Riadh Badraoui

**Affiliations:** 1 Energy and Matter Research Laboratory, National Center for Sciences and Nuclear Technologies, BiotechPole, Ariana 2020, Tunisia; 2 Department of Pharmacology and Toxicology, Unaizah College of Pharmacy, Qassim University, Buraidah 51452, Saudi Arabia; 3 Higher Institute of Applied Biology of Mednine, Route El Jorf-Km 22.5-4119 Medenine, University of Gabes, Medenine 4119, Tunisia; 4 Department of Mathematics, Physics & Statistics, University of Guyana, Turkeyen Campus, Georgetown P.O. Box 10-1110, Guyana; 5 Laboratory of General Biology, Department of Biology, University of Ha’il, Ha’il 81451, Saudi Arabia; 6 Laboratory of Histo-Embryology & Cytogenetics, Medicine Faculty of Sfax, University of Sfax, Sfax 3029, Tunisia; 7 Section of Histology-Cytology, Medicine Faculty of Tunis, University of Tunis El Manar, La Rabta, Tunis 1007, Tunisia

**Keywords:** pomegranate, irradiation, dressing, ulcer, chitosan, pharmacokinetics, stomach, healing

## Abstract

This study reported the fabrication and characterization of gastric dressing, composed of gelatine (GEL), chitosan (CH), and pomegranate peel (PP) extract. The structural changes occurring after γ-irradiation of GEL–CH–PP dressing were reported. The results showed that the electron paramagnetic resonance (EPR) spectroscopy of un-irradiated GEL–CH–PP showed two paramagnetic centers, which corresponded to g = 2.19 and g = 2.002. After irradiation, a new active centre appeared at g = 2.0035 at 10 kGy. The Fourier transform infrared spectroscopy (FTIR) analyses revealed an increase in peak intensity at C–H chains, as well as the C=O carboxyl groups at 10 kGy, due to the cross-linking phenomenon. The X-ray diffraction analysis showed a low change of crystallinity between the range of 2θ (15–30°). Moreover, γ-rays enhanced scavenging DPPH radical activity (51±%) and chelating power activities 79.12%. A significant inhibition of antibacterial and anti-biofilm activities (*p* < 0.01) was noticed. The hemolysis rates showed 0.42%, suggesting a high hemocompatibility, and exhibited significant anti-inflammatory activity in vitro (48%). In vivo, the healing effects of GEL–CH–PP dressing showed that the incidence and severity of gastric histopathological lesions decreased, compared with the ulcerated group, which could explain the bioavailability and the pharmacokinetic findings. The results highlight the loading of bioactive agents into polymer-based gastric dressings, with promising pharmacokinetics properties and effects on the induced ulcera in rabbits.

## 1. Introduction

*Helicobacter pylori* (*H. pylori*) is one of the major causes of chronic gastritis and peptic ulcers [1]. Clinically, the removal of *H. pylori* is quite challenging [2,3]. In fact, microbial resistance to current antibiotic therapies is a major cause of treatment failures and adverse clinical outcomes in gastric treatment. In this respect, the innovative download of natural compounds could be considered a promising alternative for overcoming antibiotic-resistant infection in the gastric mucosal surface. Due to its mucoadhesive qualities, chitosan (CH) is a naturally occurring antimicrobial and has wound healing properties [4,5,6,7,8] polymer that is frequently investigated for use in gastrointestinal applications. The mucosal adhesion of CH can lengthen the retention duration of drugs and facilitate their continuous penetration at the lesion site, due to the multivalent anions reaction. This drug could be other than antibiotics, such as a natural plant extract as peel pomegranate (PP) [9,10,11,12]. In fact, PP showed antibacterial and antioxidant activities, including phenolic punicalagins, gallic acid, catechin, quercetin, rutin, flavones, flavonones, and anthocyanidins [13,14,15]. Tannic acid has astringent properties, due to its ability to form macromolecular complexes with the proteins to which it binds by hydrogen links. Tannins play a protective role against gastric ulcer. However, the loaded PP extracts release rapidly. For that, utilizing encapsulation technologies for the local administration of (PP) may contribute to the development of more accurate and effective therapeutic approaches to gastric cells. GEL is being used in drug downloading because they are able to promote cell adhesion and proliferation. In fact, glycine and prolyl-hydroxyproline are the most abundant GEL-derived amino acids and peptides, respectively, in the circulation, and they might be the basis of different reactions [16,17,18] and anti-inflammatory activities [19,20]. In fact, glycine has been recognized as an anti-inflammatory agent [21]. By using GEL, the ability to adhere to the mucus layer and the intimate contact with the mucus surface will be increased, thus resulting in an increased PP extract retention time and concentration in the local sites. This would lead to an improved therapeutic effect for local diseases. In this study, the combination of CH, GEL, and the PP extract was used to produce a hybrid bioactive anti-ulcer dressing. Combining the natural advantages of CH to construct a loaded natural extract, such as PP, may be an effective strategy for treating stomach ulcers. To the best of our knowledge, there is no report on the application of such a formulation. The effect of γ-rays on these bioactive dressings, physicochemical studies, and in vitro biological analyses of the produced dressing were evaluated. The in vivo curative effect toward gastric ulcers in the prevention of acidic-induced ulcerogenesis model was determined.

## 2. Materials and Methods

### 2.1. Extraction of Gelatin

Bovine skin was soaked in 0.5 M NaOH (Merck KGaA, Darmstadt, Germany), with ratio of skin/solution at 1:5 (*w*/*v*), for 3 days to remove non-collagenous proteins. The solution was changed every day. The alkaline treated skins were washed with tap water until neutral pH. The samples were then soaked in 0.1 M citric acid (Merck KGaA, Darmstadt, Germany) at a ratio of 1:5 (*w*/*v*) for 1 h. The swollen skin was mixed with distilled water at 1:5 (*w*/*v*) at (60 °C) for 6 h. The mixtures were then filtered using filter paper to remove insoluble materials. The supernatant was freeze-dried and subjected to analyses.

### 2.2. The Gastric Dressing (CH-GEL-PP) Formulation

Briefly, CH film forming solution (1% *w*/*w*) was prepared by dissolving CH powder (Sigma Aldrich, St. Louis, MO, USA) in acetic acid solution (1%, *w*/*w*) under magnetic stirring for 8 h at room temperature (25 °C) until complete dissolution. The bovine skin GEL was dissolved in water (4%) and heated at 60 °C for 5–10 min. PP extract was dissolved in (2% *w*/*w*) ethanolic solution. Then, the prepared solutions were homogenized for 30 min.

### 2.3. Fourier Transform Infrared (FTIR)

Fourier transform infrared (FTIR) spectroscopy (Sidi Thabet, Tunisia) was used to study the structural and chemical properties of the composites. The measurements were recorded at the room temperature (25 °C) by Vertex 70 infrared spectrometer from 4000 to 400 cm^−1^ at a spectral resolution of 2 cm^−1^ and 32 scans.

### 2.4. X-ray Diffraction (XRD)

The X-ray diffraction analysis of the composites was conducted using XRD Bruker D8 (Bruker-AXS, Karlsruhe, Germany) advance with Cu-Kα radiation of wavelength λ = 1.541 Å in 2θ values in the range of 15–90°. The results obtained by X-ray measurement were analyzed with the X’PertHigh Score Plus software (version 3.0.5, PANalytical B.V., Almelo, The Netherlands).

### 2.5. Antimicrobial Activity Assay

#### 2.5.1. Microbial Strains

The antibacterial activity of GEL–CH–PP was investigated against Gram-positive *Staphylococcus aureus* (ATCC 25923), obtained from the microbiology laboratory of National center of sciences and nuclear technologies (CNSTN, Tunisia), and Gram-negative clinical strain *Helicobacter pylori*, obtained from the university hospital (laboratory of biochemistry) of Mednine, Tunisia.

#### 2.5.2. Liquid Medium Test

From a young culture on agar, a dense bacterial suspension superior to 105 Colony-forming unit (CFU) cells/mL) was prepared by dissociating three to five colonies in a 5 mL of sterile saline solution; the density of the suspension was measured using a spectrophotometer (Agilent Technologies, Santa Clara, CA, USA). The samples of different films were immersed into the saline solution containing the microorganism and stirred at room temperature for 24h. The saline solution contained about 10^6^ CFU cells/mL. The treated saline solution (0.1 mL) was inoculated in the 20 mL Mueller-Hinton agar (Sigma-Aldrich, St. Louis, Mo., USA) to cultivate the microorganisms at 37 °C for 24 h. After that, the number of colonies was counted. All glassware was sterilized in the autoclave (kalstein, Paris, France) at 120 °C for 20 min before experiments.

### 2.6. Cristal Violet Biofilm Assay

A static biofilm formation assay was performed in 96-well polystyrene plates. In brief, *H. pylori* was grown overnight in LB and diluted into fresh LB to an OD600 of 0.02. A total of 200 μL of the bacterial suspensions were mixed with 200 μL of various samples and incubated for 24 h (stationary phase) without shaking at 37 °C. The next day, LB medium was discarded, and the plates were washed three times with PBS buffer. Then, bacterial biofilms were visualized by staining with 125 μL of 0.1% crystal violet for 15 min at room temperature. Plates were then washed, and the amount of biofilm was quantified, after dissolving in 100 μL of 95% ethanol, by measuring the OD at 570 nm.

### 2.7. Antioxidant Activities

#### 2.7.1. DPPH Radical-Scavenging Activity

The scavenging of 2,2-diphenyl-1-picryl-hydrazyl-hydrate (DPPH) assays. Volume of 100 µL of sample solutions at different concentrations (0.25 to 10 mg/mL) was mixed with 100 µL of ethanol and DPPH solution (Sigma-Aldrich, St. Louis, MO, USA) (0.002%). The reaction mixture was then incubated in the dark for 60 min. The absorbance was determined at 517 nm using a 96-well microplate reader. Ascorbic acid was used as positive control. The DPPH radical scavenging activity (%) was determined using the following formula:

$$\mathrm{DPPH}\;\mathrm{radical}-\mathrm{scavenging}\;\mathrm{activity}\;\left(\%\right)=(\frac{\left[Abs_{control}-Abs_{sample}\right]}{Abs_{control}})\times 100$$

where *Abs_control_* is the absorbance of DPPH; *Abs_sample_* is the absorbance of sample.

#### 2.7.2. Ferrous Chelating Activity

A volume of 1 mL of GEL–CH–PP composite at different concentrations was added to 1 mL of distilled water and 0.05 mL of ferrous chloride solution FeCl2 (2 mM). The mixtures were incubated for 5 min at room temperature. Then, 10 µL of ferrozine solution (5 mM) was added. After a 10 min reaction time, the absorbance of each sample mixture was measured at 700 nm by a microplate reader. Ethylene diamine-tetra-acetic acid (EDTA) was employed as a positive control. The chelating power (%) was determined using the following formula:

$$\mathrm{Chelating}\;\mathrm{power}\;\left(\%\right)=(\frac{\left[A_{control}-A_{sample}\right]}{A_{control}})\times 100$$

where *A_control_* and *A_sample_* are the absorbance of the control and sample, respectively.

### 2.8. Hemolysis

The hemostatic performance of the composites was tested in vitro, as described previously [22,23]. A quantity of 800 µL of the composites at different concentrations was incubated with 200 µL of fresh goat whole blood (whole blood, normal saline = 8:10) at 37 °C for an hour. After that, the mixture was centrifuged at 4000 rpm for 5 min, and the absorbance of supernatant was measured at 545 nm. Distilled water and normal saline added with whole blood were used as positive and negative controls, respectively. The experiments were run in triplicate. Hemolysis rate (HR) was calculated as follows:

$$\mathrm{HR}=[\frac{\mathrm{AS}-\mathrm{AN}}{\mathrm{AP}-\mathrm{AN}}]\times 100$$

where AS, AP, and AN present the absorbance of the composites at different gamma doses, the positive control, and the negative control, respectively.

### 2.9. Blood Anticoagulant Index

In the present study, the blood anticoagulant index was investigated. Briefly, 100 µL of the composite at different doses were incubated with 100 mL whole blood anticoagulated by 3.8 wt.% citrate sodium (Sigma Aldrich, St Louis, MO, USA) for 1 min. A total of 25 µL of CaCl_2_ solution (Sigma Aldrich, St Louis, MO, USA) (0.1 M) was then added to each sample. Samples were taken out at indicated time points (5, 10, 30, 40, and 60 min) after the addition of CaCl_2_. After the incubation, each sample was immersed in 30 mL of deionized water, and the absorbance (AS) of the suspension was measured at 545 nm.

The blood anticoagulant index (BCI) was determined as follows:

$$\mathrm{BCI}=\left(\frac{\mathrm{AS}}{\mathrm{Aw}}\right)\times 100$$

where Aw represents the absorbance of the solution containing 100 µL whole blood and 30 mL deionized water. Then, the BCI values were plotted against the corresponding time points.

### 2.10. Evaluation of In Vitro Anti-Inflammation Activity

The effect of the different composites on heat-induced bovine serum albumin (BSA) (Sigma-Aldrich, Darmstadt, Germany) denaturation assay was carried using a method described by Chandra and coll [24]. The reaction mixtures consist of varying concentrations (100, 200, 300, and 500 μg/mL) of GEL–CH–PP, 1% *w*/*v* BSA, and phosphate buffered saline (PBS, pH 6.4) separately, while PBS was used as a control. The reaction mixtures were incubated at 37 °C for 20 min, and the temperature was increased to keep the samples at 70 °C for 5 min. After cooling, turbidity was measured at 660 nm using UV–visible spectrophotometer (Agilent Technologies, Santa Clara, CA, USA). The control represents 100% protein denaturation. The percentage inhibition of BSA denaturation was calculated as stated below:

$$\%\;\mathrm{inhibition}\;\mathrm{of}\;\mathrm{BSA}\;\mathrm{denaturation}=100\times \left[1-\left(\frac{A2}{A1}\right)\right]$$

where A1 and A2 are the absorbance of the control and the sample, respectively.

### 2.11. Drug-Likeness and Pharmacokinetics Study

Based on the ADMET (absorption, distribution, metabolism, excretion, and toxicity) properties, as previously reported, prediction of the drug-likeness and pharmacokinetics of the gastric prepared dressing was carried out [12,25,26]. Based on the physico-chemical structures, the bioavailability of GEL–CH–PP gastric dressing was also evaluated.

### 2.12. In Vivo Study Animal Model

#### 2.12.1. Rabbits Manipulation and Ethical Considerations

In this investigation, 1.5–1.7 kg New Zealand white male rabbits (of 9–10 week-old) that were produced in the Central Animal House were employed. The animals were given access to water ad libitum (Mica, Medenine, Tunisia). The animals were kept in a controlled environment with conventional temperature and humidity levels (22.2 °C and 55.5%, respectively), as well as a 12-h cycle of light and darkness. Prior to the experiment, all rabbits had a week of acclimatization. Anesthesia was induced with 10 mg/kg of ketamine (KetaminoL, Intervet International GmbH, Unterschleibheim, Germany) and 0.1 mg/kg of Xylazine (Rompun, Bayer Healthcare, Puteaux, France) [12,27]. Supplemented local anesthesia was applied after 15–20 min using 4 mg/kg carprofen (Midas Pharma, Paris, France). The rabbits were checked daily for clinical lameness or other complications. This study followed the guidelines for the care and use of laboratory animals of the National Center for Sciences and Nuclear Technologies (Tunisia), which refers to Guide for the Care and Use for Laboratory Animals, and was approved by the local Ethical committee of laboratory of Energy and Matter Research Laboratory (LR16CNSTN02).

#### 2.12.2. Induction of Gastric Ulcer and Experimental Design

The rabbits were randomly divided into 3 groups, each of which contained 8 animals: Group (I) received normal saline as control (intact stomach) (T). Groups II: acid acetic-induced gastric ulcer, in which rats received a single oral gavage dose of acid acetic gastric. The last group (III) received 30% acid acetic (0.5 mL/kg of weight) to induce stomach ulcer [28], and then they were treated with GEL–CH–PP dressing after ulceration.

#### 2.12.3. Histological Studies

All stomach samples were fixed at 10% formalin and used for histological examination by light microscopy. They were then embedded in paraffin, serially sectioned at 5 mm, and stained with hematoxylin–eosin and Giemsa stain.

### 2.13. Statistical Analyses

All experimental results are presented as means ± standard deviations (SD). Multiple comparisons were performed using analysis of variance (ANOVA), followed by Tukey’s range test. The probability value of *p* < 0.05 was considered statistically significant.

## 3. Results

### 3.1. In Vitro Biological Characterization

#### 3.1.1. Antibacterial Activities

**Figure 1:**
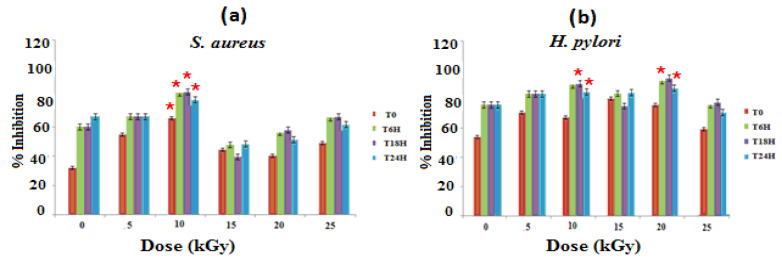
Determination of bacterial inhibitory effects of gelatine–chitosan–pomegranate peel (GEL–CH–PP) composite against *S. aureus* (**a**) and *H. pylori* (**b**) strain with different γ-radiation doses, 5, 10, 15, 20, and 25 kGy, in comparison with control samples (0 kGy). * Significant difference in the indicated group than that of the other groups at the same period of time (*p* < 0.05).

The evaluation of the antibacterial activities of the different materials on the culture medium of *S. aureus and H. pylori* strains shows that GEL–CH–PP wound dressing material inhibits bacterial growth (Figure 1). At 10 kGy, these new biomaterials showed the best antibacterial activity with an inhibition rate of 84.62% and 88.41%, respectively, against *S. aureus* and *H. pylori* after 18 h of contact in a strong synergistic effect between pomegranate peel, GEL, and CH. The inhibitory effects are not stable with the increasing of time. Gamma rays at 10 and 20 kGy demonstrated an ameliorative effect of the GEL–CH–PP dressing against pathogenic bacteria.

#### 3.1.2. Anti-Biofilm Activities

**Figure 2:**
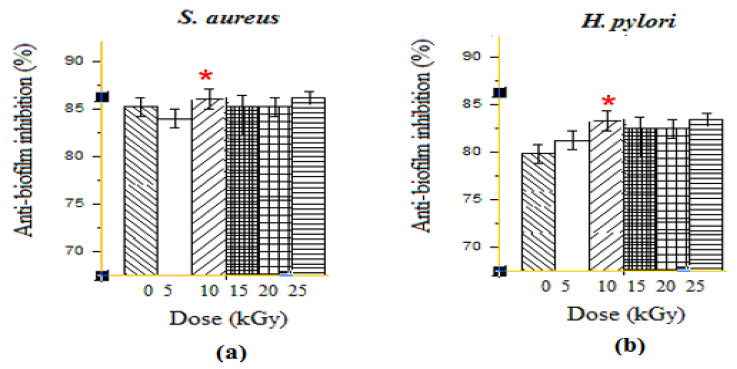
Determination of anti-biofilm activities effects of gelatine–chitosan–pomegranate peel (GEL–CH–PP) composite against *S. aureus* (**a**) and *H. pylori* (**b**) strains with different γ-radiation doses, 5, 10, 15, 20, and 25 kGy, in comparison with control samples (0 kGy). * Significant difference in the indicated group, as compared to that of 5 kGy group (*p* < 0.05).

Due to the ability of many resistant bacteria to form biofilm, the treatment against their infections is usually very appealing. Interestingly, GEL–CH–PP dressing showed very important biofilm inhibition results, which are presented in Figure 2. At the highest dose, 10 kGy, the GEL–CH–PP dressing inhibited the biofilm formation of *S. aureus* 83.84% ± 1.50 and 86.56 ± 0.60%) for *H. pylori*. The anti-biofilm activities showed a low inhibition at 15 and 20 kGy for both strains. This antibiofilm activity was not in a dose-dependent manner, and the most important inhibition was detected at 10 kGy for both *S. aureus* and *H. pylori*. The dose rate of gamma ray plays an important role in the anti-biofilm activities. In fact, the GEL–CH–PP dressing showed different behaviours and effects against bacteria biofilm.

#### 3.1.3. Antioxidant Activities

- Antioxidant activity assessed by DPPH radical scavenging test

**Figure 3:**
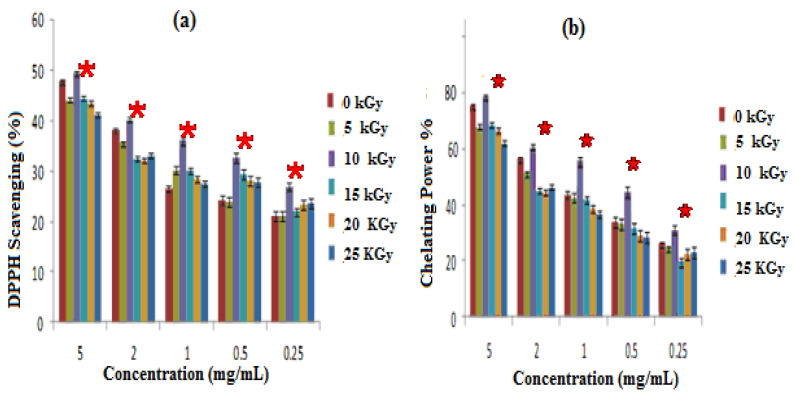
Antioxidative activities; irradiated gelatine–chitosan–Pomegranate peel (GEL–CH–PP) with different γ-radiation doses, 5, 10, 15, 20, and 25 kGy, in comparison with control samples (0 kGy): (**a**) DPPH scavenging activity; (**b**) Chelation Power on Ferrous (Fe^2+^) ions. * Significant difference in the indicated group, as compared to that of the other groups at the same concentration (*p* < 0.05).

The free radical scavenging activity of GEL–CH–PP dressings were tested using the DPPH method, and the results are shown in Figure 3a. Gamma rays were effectuated at a dose of 5 up to 25 kGy, and the scavenging ability of the composites was improved significantly (*p* < 0.05) at 10 kGy, as compared to non-irradiated samples. At this dose, the percentage scavenging activity against DPPH ranged from 31 ± 0.99 to 50 ± 1.2%. Particularly, the data showed that all the experimented composites of the samples had a significant ability to reduce the stable purple-colored radical DPPH into yellow-colored DPPH-H, which increased by 30% for concentrations corresponding to 1 mg/mL to non-irradiated samples.

- Iron-chelating ability Iron (FeII) Chelation

GEL–CH–PP dressings not having been exposed to radiation showed a more significant ability to chelate ferrous ions, in comparison with other irradiated composites. The most important chelating power was detected at 10 kGy. According to Figure 3b, the chelation power of GEL–CH–PP dressing materials showed 78.12% at 5 mg/mL. The lowest observed rate was 61.18%, detected at 25 kGy.

### 3.2. Biocompatibility

#### 3.2.1. Anti-Hemolytic Activities

**Figure 4:**
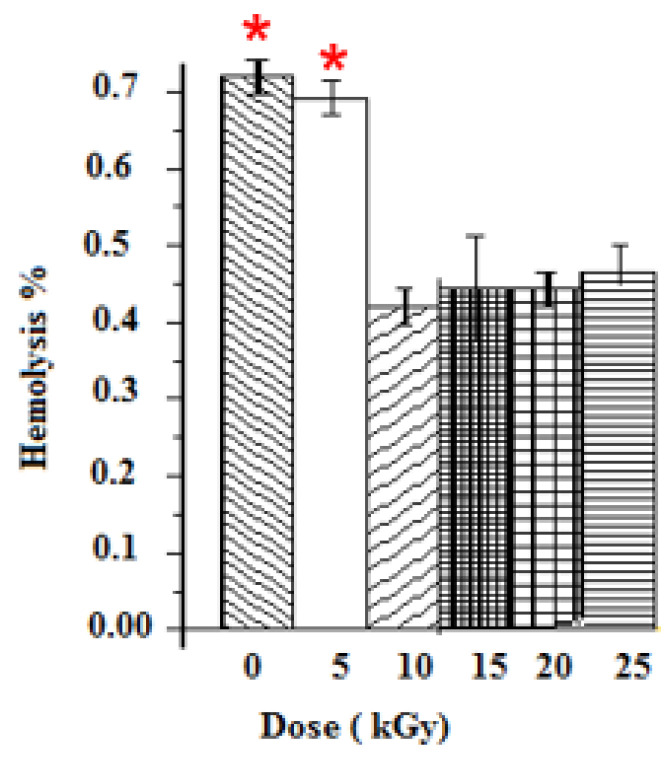
Determination of Hemolysis properties of gelatine–chitosan–Pomegranate peel (GEL–CH–PP) composite with different γ-radiation doses, 5, 10, 15, 20, and 25 kGy, in comparison with control samples (0 kGy). * Highly significant difference in the indicated group as compared to other groups (*p* < 0.05).

The anti-hemolytic activities were evaluated for five gamma ray doses. Positive and negative controls were taken with distilled water and PBS. The hemolysis was dose-dependent for all samples. Un-irradiated samples induced the highest hemolysis among samples, thus leading to the breaking of more than 0.73% of RBCs present in the solution. Samples at 10 kGy presented the lowest cytotoxic response (0.42% of hemolysis). The hemolysis rates of GEL–CH–PP dressing materials under different gamma doses were below 2% (the ideal hemolysis rate that is considered the benchmark), and no modified forms of haemoglobin were found (Figure 4).

#### 3.2.2. In Vitro Anti-Inflammation Assays

**Figure 5:**
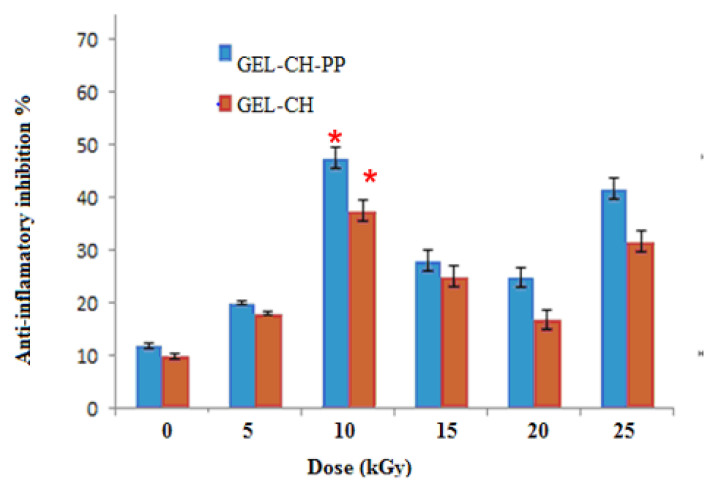
Determination of in vitro anti-inflammatory properties of gelatine–chitosan–Pomegranate peel (GEL–CH–PP) composite with different γ-radiation doses, 5, 10, 15, 20, and 25 kGy, in comparison with control samples (0 kGy). * Significant difference in the indicated group, as compared to the other groups for GEL–CH–PP (*p* < 0.01) and GEL–CH (*p* < 0.05).

In-vitro anti-inflammatory activity study was carried out at different dose of irradiation by reported method and the percentage inhibition of protein denaturation is presented in Figure 5. GEL–CH–PP gastric dressing material inhibited heat-induced BSA denaturation in a concentration-dependent manner. After irradiation, GEL–CH–PP composite significantly (*p* < 0.05) exhibited a highest inhibition of heat-induced BSA denaturation at 10 then at 25 kGy. In fact, the inhibition % of protein denaturation of these GEL–CH–PP composite was within the range of 41% to 48.0%. At 10kGy, the anti-inflammatory activity augmented approximately 5 times of those un-irradiated samples.

### 3.3. Physico-Chemical Analyses

#### 3.3.1. Electron Paramagnetic Resonance Analysis (EPR)

**Figure 6:**
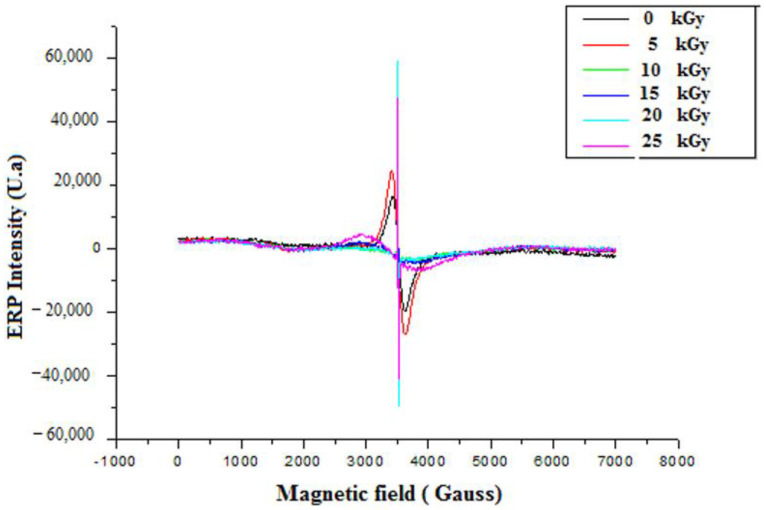
EPR spectrum of irradiated gelatine–chitosan–Pomegranate peel (GEL–CH–PP) composite with different γ-radiation doses, 5, 10, 15, 20, and 25 kGy, in comparison with control samples (0 kGy).

Before irradiation, EPR measurements of GEL–CH–PP dressing gave two signals centred at g1 = 2.19 and g2 = 2.002 and a line width (peak-to-peak) of approximately Δpp = 200 G (Figure 6). The doses 0 and 5 kGy were approximately superposed. Whereas 10, 15, 20, and 25 were superposed at around 3481 G, and g3 = 2.0035 were probably related to peroxide radicals [29]. The radical amount was proportional to the absorbed dose. From the present spectrum, it can be seen that the intensity of the peaks increased with increasing dose. This confirms the augmented number of free radicals at higher doses.

#### 3.3.2. Fourier Transform Infrared (FTIR)

**Figure 7:**
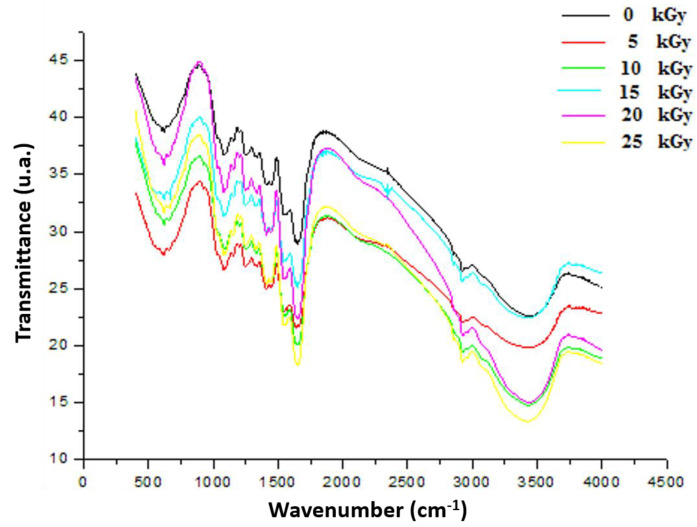
FTIR spectrum of irradiated gelatine–chitosan–Pomegranate (GEL–CH–PP) composite of functional groups with different γ-radiation doses, 5, 10, 15, 20, and 25 kGy, in comparison with control samples (0 kGy).

As shown in Figure 7, the peak at 3420 cm^−1^ in the FTIR spectra was attributed to OH and NH stretching vibrations originating from GEL, CH, and PP extract. GEL comprises a protein molecule bound to a polymer of amide groups, OH and NH [30]. The symmetric or asymmetric stretching of aliphatic CH and CH2 group contained in fatty acids, identified in the PP extract, was attributed to the peak characteristics of 2927 cm^−1^ and 2851 cm^−1^ [31]. The CO strong peaks matched the 1800 and 1600 cm^−1^ band seen in protein structures. The band attributable to the C=O group, which was an aliphatic ketone molecule, showed at wavelengths between 1750 and 1625 cm^−1^. The band at 1538 cm^−1^ had been assigned to the aromatic CH group band. The occurrence of a peak at 1587 cm^−1^ could be caused by amide group CN stretching or NH deformation [32]. The peak at 1053 cm^−1^ after treatment of the dressing with various gamma rays could be attributable to the presence of adsorbed particles on the surface of the PP extract. These spectra, attributed to band shift phenomena, are frequently interpreted as the manifestation of gradual changes in the IR frequency associated with a specific chemical bond after gamma rays under the influence of molecular interactions between GEL, CH, and PP extracts after irradiation.

#### 3.3.3. The X-ray Diffraction (XRD)

**Figure 8:**
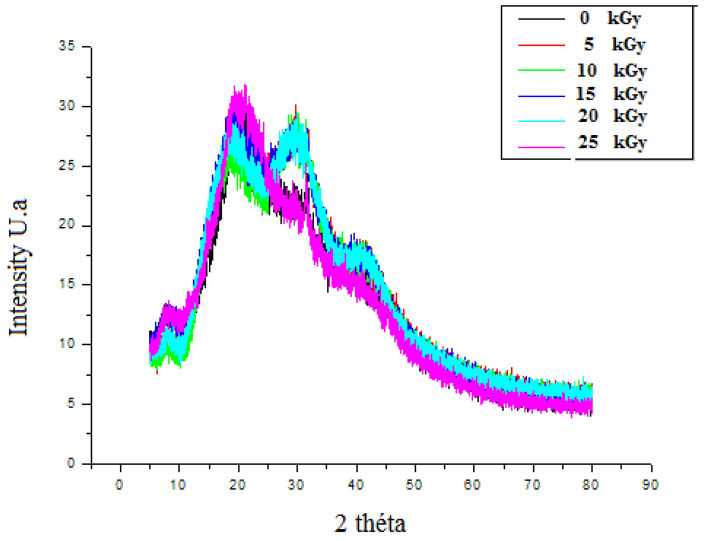
X-ray diffractograms of irradiated gelatine–chitosan–Pomegranate peel (GEL–CH–PP) composite of with different γ-radiation doses, 5, 10, 15, 20, and 25 kGy, in comparison with control samples (0 kGy).

The XRD patterns of un-irradiated dressing exhibited a band of low intensities, which emphasized the semi-amorphous nature of the material. The peak emerging at 2θ = 20.9° had clearly confirmed the CH molecule existence [33]. GEL powder showed an amorphous morphology with a characteristic broad hump in the range of 15–30. 2θ. Moreover, a sharp peak with low intensity was located at 2θ = ~7°. These characteristic peaks are usually assigned to the triplehelical crystalline structure in GEL [34]. A detected broad peak at around 2θ, equaling 10°, was attributed to the PP extract [35]. After irradiation, the crystallization behavior revealed that, up to 20 kGy, the original ordered atomic arrangement of the bioactive dressing was unaffected or unbroken by the gamma rays. However, after 25 kGy, the intensity of the crystalline sharp peaks of the prepared dressing at around 30^o^ was reduced. It might be attributed to the interaction between crystalline nature of PP particles and amorphous polymer chains (Figure 8).

### 3.4. Druggability and Pharmacokinetics of Gastric Dressing GEL–CH–PP

**Figure 9:**
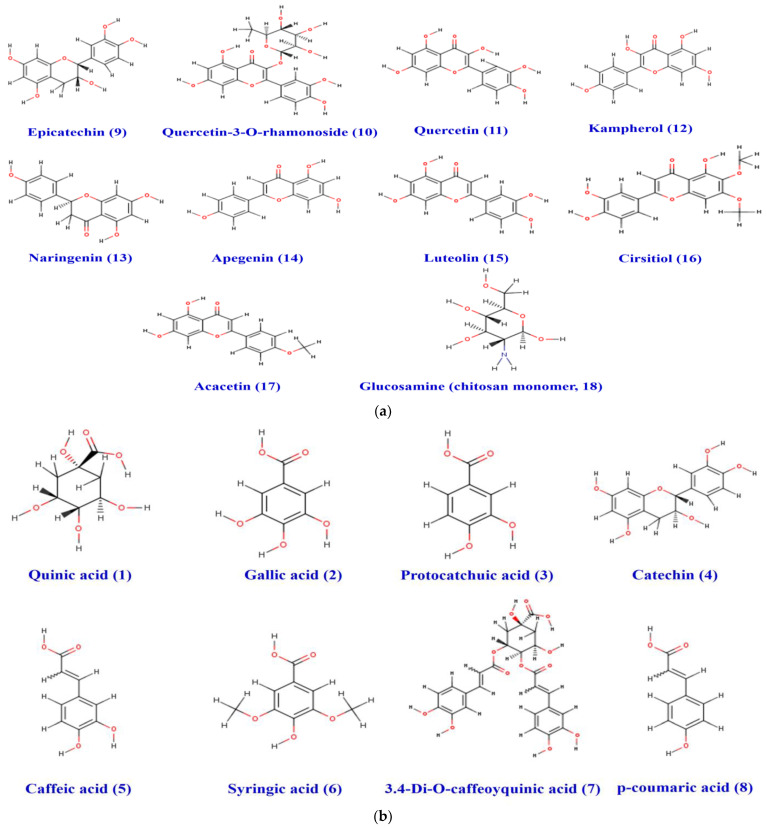
(**a**) Chemical structure of the acidic phenols (1–8) identified in the pomegranate fruit peel. (**b**) Structure of the different flavonoids (9–18) and chitosan monomer identified in the pomegranate fruit peel.

**Figure 10:**
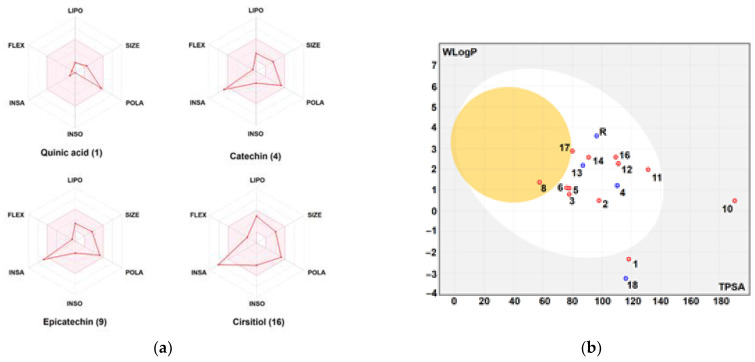
Bioavailability polygons: (**a**) and the boiled-egg model; (**b**) of the different phytochemicals identified in the pomegranate fruit peel. FLEX: flexibility, INSA: insaturation, INSO: insolubility, LIPO: lipophilicity, POLA: polarity, SIZE: molecular size. Good oral bioavailability (in the pink area, A), high GI absorption and BBB permeation (white and yellow areas in B, respectively). Red and blue dots, represent interaction with P-glycoprotein (PGP+ and PGP-, respectively).

The physico-chemical characteristics, lipophilicity, druggability, and pharmacokinetic attributes of pomegranate fruit peels identified the compounds. Most of the compounds met Lipinski’s rule and had acceptable bioavailability scores (BAS), as assessed by the bioavailability polygons (Figure 9a,b) and the boiled-egg model (Figure 10a,b). All BAS values were positive and ranged between 0.11 and 0.85. Gastrointestinal absorption (GI) and blood–brain barrier (BBB) permeation have been also studied. The majority of the compounds possessed high GI absorption and low BBB permeation (Table 1) and were not a substrate of P-glycoproteins. Inhibition of cytochrome P450 (CYP) enzymes, particularly the CYP3A4, CYP2D6, CYP2C19, CYP2C9, and CYP1A2 isoforms, was carried out for the PP extract phytocompounds. CYP2C19 was not inhibited by all the compounds. A1, A2, A4-A9, F1, and F3 inhibited none of the studied CYP isoforms. PP extract compounds showed low-to-moderate skin permeability.

### 3.5. In Vivo Study

#### Effect of GEL–CH–PP Dressing on Acid Acetic-Induced Histopathological Alterations

**Figure 11:**
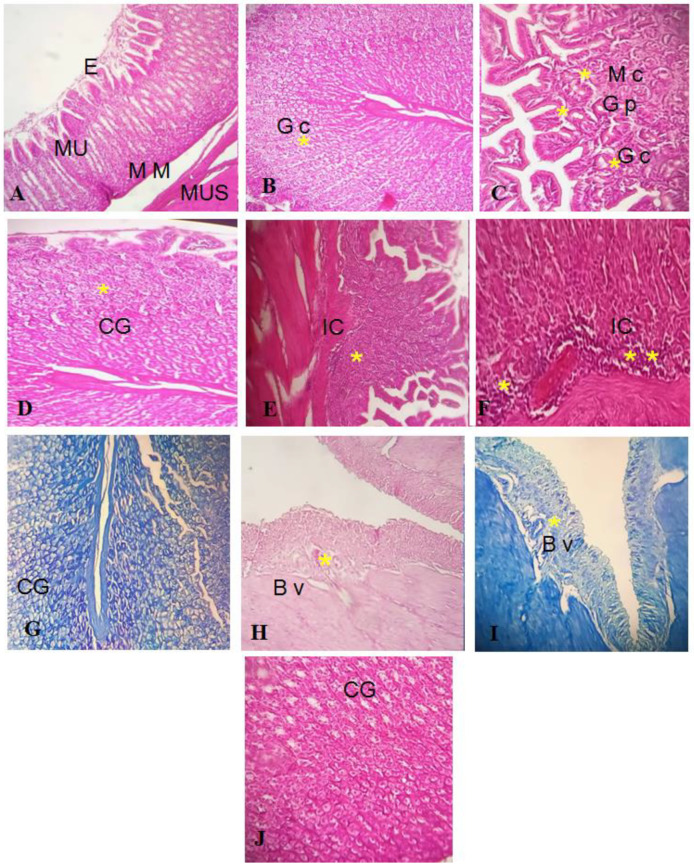
((**A**–**C**): control group I) Normal stratification of stomach layer. (**E**): epithelium, Mu: mucosa, MM: muscularis mucosa, Mus: muscularis. (**B**): Gc: glandular cells, (**C**): PG: pyloric gland, M c: mucosis cells, GC: glandular cells; ((**D**–**F**): group treated with acetic acid II) normal stratification, acidic acid did not alter stomach layer, but apparition of cells inflammatory. GC: glandular cells. IC: inflammatory cells ((**G**–**J**) group after GEL–CH–PP treatment III), GC: glandular cells. BV: blood vessels. Hematoxylin-Eosine and Giemsa strain. Note the histo-pathological features (severe bleeding, inflammatory cell infiltration, epithelial cell loss, submucosal edema, and vascular congestion), which are marked by asterisks.

Histopathological investigation validated the antiulcer activity of the GEL–CH–PP dressing, as seen in the gross findings. The gastric mucosa in the control and GEL–CH–PP dressing groups had normal architecture (Figure 11A–C). However, acid acetic-treated rabbits’ stomach mucosas displayed substantial histological alterations, including severe bleeding, inflammatory cell infiltration, epithelial cell loss, submucosal edema, and vascular congestion (Figure 11D–F). When compared to the acid acetic ulcerated stomach rabbits, the incidence and severity of stomach histological lesions decreased. From the Geimsa stain, no *H. Pylori* colonisation was identified (Figure 11G–J).

## 4. Discussion

Gastric ulcer is one of the major problems that may be attributed to the exposure of stomach mucosa to various damaging factors. In this respect, natural-loaded bioactive substances have been extensively investigated recently. 

In this study, EPR spectroscopy detects paramagnetic substances, such as radicals from 5 up to 25 kGy. The signal intensity reflected the total absorbed energy of samples under resonance conditions (i.e., the signal intensity was in direct proportion to the number of radicals in the samples). The formation of these radicals can be explained by the fact that the GEL–CH–PP dressing contains alcohol groups, glycosidic, acetal oxygen groups, carboxyl, amino, hydroxyl groups, and carbonyl groups. The FTIR analysis confirmed that the loaded bioactive dressing exhibited no spectral changes. The results confirmed that, after irradiation, PP was physically entrapped (encapsulated) within both GEL and CH. In fact, no spectral shift was found to be attributed to a chemical interaction between PP, GEL, and CH. The incorporation of PP extract resulted in a significant increase in the intensity of C–H stretching bands at 3410, 2850, 1550, 1409, and 1080 cm^−1^, reflecting the successful integration. The existence of various phenolic compounds explains the ability to react with DPPH and chelate iron differently. Caffeic acid, gallic acid, and protocatechuic acid have significant DPPH scavenging actions because they have two-to-three hydroxyl groups, which is why they have high antioxidant activity. The antioxidant activity of phenolic acids depends, in part, on the amount of hydroxyl groups in the molecule [36]. Gallic acid’s high scavenging ability is attributed to the presence of electron-donating or high electron density hydroxyl groups at the meta and para locations [37]. This acid molecule contains the carboxylic acid O–H stretch attributed to around to 2900 cm^−1^ and is also characterized by the carbonyl stretch C=O of a carboxylic acid that appears as an intense band at 1690 cm^−1^, as confirmed in the present study by FT-IR analysis. One study demonstrated that p-coumaric reacts poorly with DPPH and that it has a limited capacity to donate hydrogen [37,38]. Concerning ferrous chelating activity, the present formulated composite enclosed catechol or galloyl groups, such as caffeic acid. This latter compound contains hydroxyl functional groups on the benzene ring and an unsaturated bond of its ethylenic side chain identified by FTIR at 720 cm^−1^, considered as important sites for reactions with reactive oxygen species. The antioxidative actions are affected by the number and position of hydroxyl groups bound to the aromatic ring, the binding site, the mutual location of hydroxyl groups in the aromatic ring, and the type of substituents and their factors. Ortho-dihydroxyphenols, including caffeic acid, were shown to have the highest antioxidant effectiveness. The ability of this phenolic acid group to donate electrons is what gives it its activity [39,40,41,42]. According to one study, the third OH group in gallic acid stabilizes the process of electrical delocalization inside the ring structure [43]. Additionally, it was discovered that substituting with a hydroxyl group, rather than a methoxy group, was more efficient [44]. Thus, the in vitro evaluation of GEL–CH–PP dressing occurred firstly through antioxidant activities after irradiation. At 10 kGy, the bioactive dressing showed the strongest DPPH scavenging activity and iron chelating capacity. The therapeutic activity was due to the existence of PP extract, including phenolic punicalagins, gallic acid and other fatty acids, tannins catechin, quercetin, rutin, flavones, flavonones, and anthocyanidins. These findings support an earlier study that suggested irradiating biological material up to a dosage level of 12 kGy increased the DPPH^•^ scavenging activity [45,46]. In fact, one study confirmed that the tannins found in PP extract can prevent stomach ulcers. It functions by raising the quantity of adherent and free mucus secreted by the stomach wall [47]. This may reduce the amount of oxygen-derived free radicals produced.

Based on their antioxidant activity, as well as their antibacterial capabilities, some active ingredients with ulcer-protective characteristics have been discovered. Here, it is possible to infer that CH can bind to the DNA of *E. coli* and *S. aureus* microbes at a concentration of 10 kGy. Additionally, CH can chelate metals, inhibit spore elements, and bind to crucial nutrients for microbial growth. It can also penetrate through the bacterial cell wall [48]. Particularly, the produced bioactive dressing combats *H. pylori,* which produces urease and secretes it to survive in the stomach’s acidic environment. *H. pylori* colonizes the stomach mucosa and selectively clings to its cell-cell junctions. The antimicrobial action of PP is related to the polyphenolic tannins, particularly punicalagin and ellagic acid content, in the extract [49]. Furthermore, it has been claimed that polyphenols can harm the microbial respiratory chain by reducing oxygen consumption and so restricting the oxidation of NADH. Since microorganisms behave differently in biofilm environments, some hypothesized mechanisms for polyphenols’ biofilm eradication and formation inhibitory effects have been proposed. Protein precipitation and enzyme inactivation are the mechanisms by which PP extracts prevent biofilm growth and biofilm formation [50]. However, these mechanisms have not yet been verified. Interestingly, this later compound also affects the microbiota’s composition and promotes the growth of good bacteria, such as *Bifidobacterium* and *Lactobacillus*, while inhibiting the growth of strains of *Bacteroides, clostridium,* and *Enterobacteriaceae* [51]

As shown in the present research, after dressing treatment, the Giemsa coloration did not detect the presence of *H. pylori*, and the bacteria-associated inflammatory infiltration was attenuated. The repair effect of GEL–CH–PP treatment for a stomach ulcer was evaluated by H&E staining. The stomach of the ulcerated animals did not show an accentuate erosion of gastric mucosa, but neutrophil infiltrations were found. The stomach mucosal layer of the wound was repaired completely after the administration of GEL–CH–PP gastric dressing, which may be due to abundant pharmacological activities of the PP extract supporting tissue repairing. These results prove that the GEL–CH–PP gastric dressing can be used as a high-performance sealant for the treatment of perforated injures in the deep area of the body.

On the other hand, determining the hematocompatibility of GEL–CH–PP sample, the assessment of haemolytic activity toward RBCs was effectuated. CH is a hydrophilic polymer that is rapidly degradable via human enzymes, which results in biocompatibility. GEL is an extremely hydrophilic substance. Moreover, the different extracts of PP exhibited no hemolytic activity, ensuring the biosafety of these extracts. Inflammatory phenomena are connected to the hemolytic process. In fact, it results in the production of certain red cell constituents, known as cell damage-associated molecular patterns (DAMPs), which can cause an inflammatory response. Herein, the anti-inflammatory potential of GEL–CH–PP dressing using an in vitro inflammation model was evaluated. In fact, denatured proteins (DP) are considered one of the inflammatory mediators. Therefore, agents that limit the formation of DP aggregates and protein condensation are helpful for treating the disorders that arise during a chronic inflammatory response [12,52,53,54,55]. In our current findings, GEL–CH–PP dressing exhibited a high inhibition of heat-induced protein. In fact, the anti-inflammatory effect of PP on an in vitro gastric model demonstrated that the total tannins and punicalagin can inhibit the AKT activity, NF-KB activation, and COX-2 expression induced by TNF α [25,54,55,56]. One research demonstrated that pre-treatment with a polyphenolic aqueous extract from PP extract can reduce many inflammatory mediators [52,57]. This action was mostly caused by ellagic acid; however, other ellagitannins may have individually contributed to the mixture’s biological activity. These data indicate that, mainly at 10 kGy, GEL–CH–PP could be considered an anti-inflammatory composite.

The physico-chemical properties, lipophilicity, druggability, and pharmacokinetic attributes for the 17 different phytochemicals of the PP extract, together with CH monomer and esomeprazole as reference compounds, were determined. Previous reports showed that the assessment of these parameters is important in the drug design, management, and avoidance of any drug failure in the late stages [25,26,52,58]. As shown, the majority of the PP compounds met the Lipinski’s rule (except compounds A8 and F3). The bioavailability scores (BAS) of all the compounds confirmed their physiological activities and the possible oral administration. This was also supported by the bioavailability polygons. All the BAS values ranged from intervals of inactive to moderately active. As gastrointestinal absorption (GI) and blood–brain barrier (BBB) permeation are two essential pharmacokinetic parameters, they have been assessed. Most of the compounds possessed high GI absorption, but low BBB permeation, which was confirmed by the boiled-egg model [26,55]. These attributes have been reported to be directly linked to the 3D structures of the compounds [12,25,52,55]. Most of the compounds were predicted as not being substrates of P-glycoproteins that indicates the absence of any disruption in drug transport [58,59]. The inhibition of cytochrome P450 (CYP) enzymes, particularly the CYP3A4, CYP2D6, CYP2C19, CYP2C9, and CYP1A2 isoforms, was carried out for the PP phytocompounds. CYP2C19 was not inhibited by all the compounds. A1, A2, A4–A9, F1, and F3 inhibited none of the studied CYP isoforms, which indicates low-to-no disruption of drug metabolism [25,55,58]. Using both the molecular weight and lipophilicity of PP compounds, the skin permeability values (Kp) were explored. Our results denoted low-to-moderate skin permeability [12,55,59,60]. Taken together, the druggability and the pharmacokinetic properties results may justify the in vivo and the in vitro findings of the current study. Clinically, both of the scaffold-based GEL [22] and CH molecules [61] exhibited skin ulcer healing. However, the treatment of gastric ulcers has not yet been carried out. Concerning the PP extract gel, clinically, the composite was administered to patients and compared to that of a placebo gel. The PPE gel was significantly effective in reducing the pain, ulcer size, and healing duration of ulcers over a period of one week in the management of aphthous ulcers [61].

Our results provided evidence for the gastro-curative activity of GEL–CH–PP in a rabbit model. As a promising antiulcer phytomedicine, the natural bioactive dressing could maintain gastric mucosal integrity, and we should discuss the results and how they can be interpreted from the perspective of previous studies and of the working hypotheses. The findings and their implications should be discussed in the broadest context possible. Future research directions may also be highlighted.

## 5. Conclusions

In conclusion, this study showed that GEL–CH–PP composite, a new natural polymer loading with PP extract, can act as anti-ulcer drugs. Using Cobalt-60, at a dose of 10 kGy, the composite showed the highest antibacterial, anti-biofilm, and anti-oxidative activities. Additionally, this research indicated that this bioactive gastric dressing is an excellent candidate for reducing inflammatory and haemolytic responses. The GEL–CH–PP composite was determined to have appropriate drug-like molecules with acceptable pharmacokinetic characteristics. The in vivo rabbit research confirmed that the GEL–CH–PP composite accelerated the healing of gastric lesions. This finding provides a new approach to ulcer therapy.

## Figures and Tables

**Table 1:** Physico-chemical properties, lipophilicity, druggability, and pharmacokinetic attributes of the different phytochemicals identified in the pomegranate fruit peel (1–17), chitosan monomer (glucosamine) (18), and esomeprazole (19) as reference compounds.

Entry	1 (A1)	2 (A2)	3 (A3)	4 (A4)	5 (A5)	6 (A6)	7 (A8)	8 (A9)	9 (F1)	10 (F3)	11 (F6)	12 (F7)	13 (F8)	14 (F9)	15 (F10)	16 (F11)	17 (F12)	18 (Chito-san)	19 (Ref)
	Physicochemical properties																		
Molecular weight (g/mol)	192.17	170.12	154.12	290.27	180.16	198.17	516.45	164.16	290.27	448.38	302.24	286.24	272.25	270.24	286.24	330.29	284.26	179.17	345.42
Solubility/Lipophilicity/Druggability																			
Lipinski	Yes	Yes	Yes	Yes	Yes	Yes	No	Yes	Yes	No	Yes	Yes	Yes	Yes	Yes	Yes	Yes	Yes	Yes
Biovailability Score	0.56	0.56	0.56	0.55	0.56	0.56	0.11	0.85	0.55	0.17	0.55	0.55	0.55	0.55	0.55	0.55	0.55	0.55	0.55
Pharmacokinetics																			
GI Absorption	Low	High	High	High	High	High	Low	High	High	Low	High	High	High	High	High	High	High	Low	High
BBB Permeant	No	No	No	No	No	No	No	Yes	No	No	No	No	No	No	No	No	No	No	No
P-gp Substrate	No	No	No	Yes	No	No	Yes	No	Yes	No	No	No	Yes	No	No	No	No	Yes	Yes
CYP1A2 inhibitor	No	No	No	No	No	No	No	No	No	No	Yes	Yes	Yes	Yes	Yes	Yes	Yes	No	Yes
CYP2C19 inhibitor	No	No	No	No	No	No	No	No	No	No	No	No	No	No	No	No	No	No	Yes
CYP2C9 inhibitor	No	No	No	No	No	No	No	No	No	No	No	No	No	No	No	Yes	Yes	No	No
CYP2D6 inhibitor	No	No	No	No	No	No	No	No	No	No	Yes	Yes	No	Yes	Yes	Yes	Yes	No	Yes
CYP3A4 inhibitor	No	No	Yes	No	No	No	No	No	No	No	Yes	Yes	Yes	Yes	Yes	Yes	Yes	No	Yes
Log Kp (cm/s)	−9.15	−6.84	−6.42	−7.82	−6.58	−6.77	−8.37	−6.26	−7.82	−8.42	−7.05	−6.70	−6.17	−5.80	−6.25	−6.14	−5.66	−9.88	−6.82

Acidic phenols (A; 1–8), Flavonoids (F; 1–8), Gastro-intestinal (GI) absorption, Blood–brain barrier (BBB), P-glycoprotein (P-gp)

## Data Availability

Data is contained within the article.
